# Hemorrhagic Stroke in a 24-Year-Old Male With Polycythemia Vera: A Case Report and Literature Review

**DOI:** 10.7759/cureus.76294

**Published:** 2024-12-24

**Authors:** Josephine M Rivera, Paul Vincent Opinaldo

**Affiliations:** 1 Adult Neurology, Quirino Memorial Medical Center, Quezon City, PHL; 2 Neurology, St. Luke's Medical Center, Quezon City, PHL

**Keywords:** hemorrhagic stroke, intracerebral hemorrhage, neurology and critical care, polycythemia vera, stroke

## Abstract

Stroke is the second leading cause of death worldwide, according to the latest report by the World Health Organization (WHO). Intracerebral hemorrhage comprises 20-25% of the stroke in the young, with incidence rates of three to six in 100,000 people per year. One of the most common and important causes of hemorrhagic stroke in the general population is hypertension. Polycythemia vera (PV) can manifest with both cranial thrombosis and hemorrhage. We report a 24-year-old male, known hypertensive, who presented as a pontine hemorrhage. Further examination with imaging and laboratory tests demonstrated a diagnosis of PV. In PV, platelet dysfunction and acquired Von Willebrand syndrome are predisposing factors to bleeding. Associated blood viscosity and the proinflammatory state cause vascular remodeling, leading to hypertension. These mechanisms have collectively been described to cause hemorrhagic stroke in PV.

## Introduction

The overall incidence of all strokes in the young, both ischemic and hemorrhagic, ranges from seven to 15 in 100,000 people per year. In those younger than 35 years old, the rates are less than 10 in 100,000 people/year. Intracranial hemorrhage comprises 20-25% of the stroke in the young, with incidence rates of three to six in 100,000 people per year. Stroke is the second leading cause of death worldwide, according to the latest report by the World Health Organization (WHO) [1]. The burden of this illness does not only lie in its high rate of mortality but also resides in the poor quality of life and disability left in some of its survivors. Stroke in the young has been commonly defined as stroke in adults younger than 45 years old [2]. Polycythemia vera (PV) is a form of chronic myeloproliferative neoplasm (MPN) and is the most common form of MPN, wherein there is an increase in red cell production and substantial morbidity and mortality from venous and arterial thrombosis, which may cause cerebral infarction [3]. In some cases, PV also presents with a hemorrhagic type of stroke due to platelet dysfunction. In approximately 15% of individuals with PV, the disease may present first with stroke [4]. Among these, a hemorrhagic type of stroke occurs more frequently due to platelet dysfunction.

## Case presentation

**Figure 1:**
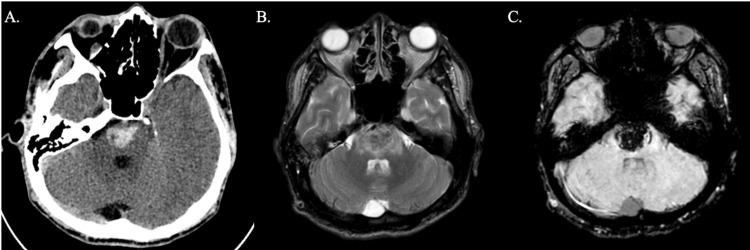
(A) Plain cranial CT scan axial view demonstrating an acute pontine hemorrhage measuring 1.7 × 1.9 × 1.7 cm amounting to approximately 2.8 cc. Cranial MRI with contrast (B) axial view T2 and (C) axial view GRE sequences demonstrating a subacute pontine hemorrhage.

This is a case of a 24-year-old male, known hypertensive, who was noncompliant with medications and a smoker for five years, consuming 10 sticks a day, and presented with sudden right-sided weakness of 11 hours duration. On examination, the initial BP was 150/100 mmHg; the patient was awake, was able to follow commands, responded through nodding and hand gestures, and was anarthric with bilateral central facial palsy and with right-sided weakness graded as 1/5 of both right upper and lower extremities. A plain cranial CT scan was done, which showed a hemorrhage in the pons amounting to approximately 2.8 cc (Figure 1A). A cranial MRI and MR angiography with contrast were done, revealing a subacute pontine hemorrhage and an unremarkable MR angiography (Figures 1B, 1C).

**Figure 2:**
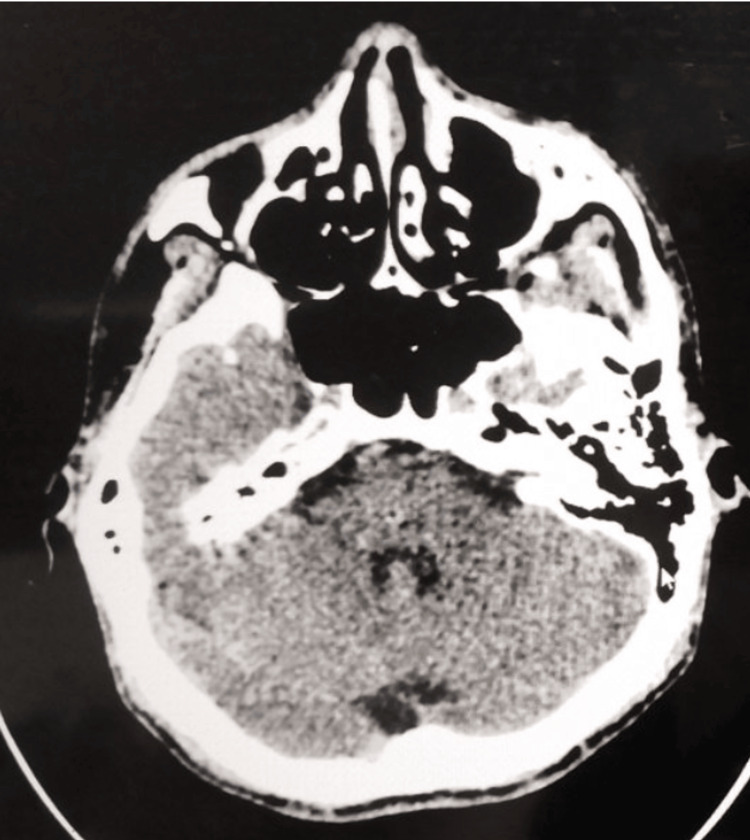
Plain cranial CT scan axial view on day 28 post-ictus, demonstrating resolution of the pontine hemorrhage.

The patient’s initial complete blood count during the admission showed a hemoglobin of 20.3 g/dL, hematocrit of 62%, WBC of 9.0 × 109/L, neutrophils of 0.81, lymphocytes of 0.14, and a platelet count of 266 × 109/L. A diagnosis of PV was considered, and further workup was done. The JAK-2 mutation analysis was negative, and serum erythropoietin level was low at 1.9 mIU/mL (normal value, 2.59-18.5). The patient was diagnosed with PV based on the 2016 revised WHO diagnostic criteria for PV and essential thrombocythemia. Other diagnostic tests for stroke in the young and secondary causes of hypertension were done, such as renal function, thyroid function test, urine drug test, and whole abdominal CT scan, which were all unremarkable. The patient was managed as a case of a stroke in the young, presenting as a pontine hemorrhage secondary to hypertension and PV. The patient stayed in the intensive care unit for several days and demonstrated clinical improvement with medical and supportive management, which included adequate hydration, phlebotomy, and management of the complications and deficits associated with his stroke. Aspirin 80 mg once a day was started when the repeat plain cranial CT scan was done on day 28 post-ictus, which showed complete resolution of the pontine hemorrhage (Figure 2). The patient was discharged after 29 days. The patient could swallow without dysphagia and was able to respond and communicate more comprehensibly, with improved bilateral facial palsy and better motor strength in the right extremities.

## Discussion

PV was first described in 1982 and has an incidence of 2.5-10 per 1,000,000 people. It is the most common form of MPN, wherein there is an increase in red cell production [3]. In the central nervous system manifestations of PV, intracranial thrombosis is more common than hemorrhage [3,5]. Here, we discuss a case of PV presenting as intracranial hemorrhage.

In the 2016 revised WHO diagnostic criteria of PV and essential thrombocythemia, there are three major criteria and one minor criterion. The three major criteria are as follows: (1) hemoglobin level >16.5 g/dL in men and >16 g/dL in women or hematocrit >49% in men and >48% in women, (2) bone marrow tri-lineage proliferation with pleomorphic mature megakaryocytes, and (3) presence of JAK2 mutation. The minor criterion is a subnormal serum erythropoietin level. A diagnosis of PV requires all three major criteria or two major criteria with one minor criterion. However, a bone marrow biopsy is not needed in the presence of hemoglobin >18.5 g/dL or hematocrit level of 55.5% in men and hemoglobin >16.5 g/dL or hematocrit of 49.5% in women [6]. In the case presented, the initial hemoglobin was 20.3 g/dL, hematocrit was 62%, JAK-2 mutation analysis was negative, and serum erythropoietin level was low at 1.9 mIU/mL. Approximately 5% of PV will present with a negative JAK-2 mutation analysis [7]. There are multiple factors linked to the cause of bleeding diathesis in PV. Some literature would show evidence of an acquired Von Willebrand syndrome occurring in PV, whereas others would implicate platelet dysfunction [6,8]. PV is also associated with an increase in the prevalence of hypertension [5]. This was attributed to the increase in blood viscosity due to elevated hematocrit levels in PV. There are several other postulated mechanisms that could explain the physiology behind hypertension caused by PV. In PV, there is an increase in cell turnover, an increase in fibrinogen levels, a heightened inflammatory state, and an increase in plasma volume. Collectively, all these events lead to hypertension. It was concluded that there is a direct effect between blood viscosity brought about by an increase in hematocrit level and hyperfibrinogenemia and elevated blood pressure [7]. A proinflammatory state has also been observed in PV. This inflammation causes endothelial dysfunction and contributes to vascular remodeling, atherosclerosis, and a decrease in vascular compliance, contributing to the pathophysiology of hypertension [9]. A study by Letcher et al. showed that there is an abnormal increase in blood viscosity in patients with untreated hypertension [10]. The increase in blood viscosity adds to the overall increase in the total peripheral resistance [10]. According to Cinar et al., an increase in the blood viscosity of 25% decreases the blood flow rate by 20%, which increases the blood pressure by 25% [11].

The main goal of therapy for PV is to prevent its complications of thrombosis and hemorrhage. It is recommended to keep the hematocrit level below 45%, with regular phlebotomy and aspirin as the treatment of choice if there are no contraindications. Cytoreductive therapy is recommended for high-risk PV patients, and the drug of choice is hydroxyurea [6]. The patient is on regular follow-up with repeat complete blood count check and phlebotomy if warranted and a possibility to start hydroxyurea if needed.

## Conclusions

This report demonstrates that although not commonly reported, PV is also a risk factor for hemorrhagic stroke in young patients. Multiple factors have been demonstrated to contribute to the development of hemorrhagic stroke. This includes platelet dysfunction and acquired Von Willebrand syndrome predisposing to bleeding and increased blood viscosity and the proinflammatory state leading to vascular remodeling and subsequently causing hypertension. Therefore, prompt diagnosis and management of PV and its complications are necessary.
