# Correction: Hemorrhagic Stroke in a 24-Year-Old Male With Polycythemia Vera: A Case Report and Literature Review

**DOI:** 10.7759/cureus.c208

**Published:** 2025-01-27

**Authors:** Josephine M Rivera, Paul Vincent Opinaldo

**Affiliations:** 1 Adult Neurology, Quirino Memorial Medical Center, Quezon City, PHL; 2 Neurology, St. Luke's Medical Center, Quezon City, PHL

This article has been corrected to fix a typo in the first sentence of the Discussion section:

- Original: "PV was first described in 1982 and has an incidence of 2.5-10/1000/000** **people."
- Corrected: “PV was first described in 1982 and has an incidence of 2.5-10 per 1,000,000 people.”

